# Virus-like particle production with yeast: ultrastructural and immunocytochemical insights into *Pichia pastoris *producing high levels of the Hepatitis B surface antigen

**DOI:** 10.1186/1475-2859-10-48

**Published:** 2011-06-26

**Authors:** Heinrich Lünsdorf, Chandrasekhar Gurramkonda, Ahmad Adnan, Navin Khanna, Ursula Rinas

**Affiliations:** 1 Helmholtz Centre for Infection Research (VAM), Braunschweig, Germany; 2 Helmholtz Centre for Infection Research (SB), Braunschweig, Germany; 3 International Centre for Genetic Engineering & Biotechnology, New Delhi, India; 4 Department of Chemistry, Government College University Lahore, Pakistan; 5 Leibniz University of Hannover, Technical Chemistry - Life Science, Hannover, Germany

## Abstract

**Background:**

A protective immune response against Hepatitis B infection can be obtained through the administration of a single viral polypeptide, the Hepatitis B surface antigen (HBsAg). Thus, the Hepatitis B vaccine is generated through the utilization of recombinant DNA technology, preferentially by using yeast-based expression systems. However, the polypeptide needs to assemble into spherical particles, so-called virus-like particles (VLPs), to elicit the required protective immune response. So far, no clear evidence has been presented showing whether HBsAg assembles in vivo inside the yeast cell into VLPs or later in vitro during down-stream processing and purification.

**Results:**

High level production of HBsAg was carried out with recombinant *Pichia pastoris *using the methanol inducible *AOX1 *expression system. The recombinant vaccine was isolated in form of VLPs after several down-stream steps from detergent-treated cell lysates. Search for the intracellular localization of the antigen using electron microscopic studies in combination with immunogold labeling revealed the presence of HBsAg in an extended endoplasmic reticulum where it was found to assemble into defined multi-layered, lamellar structures. The distance between two layers was determined as ~6 nm indicating that these lamellas represent monolayers of well-ordered HBsAg subunits. We did not find any evidence for the presence of VLPs within the endoplasmic reticulum or other parts of the yeast cell.

**Conclusions:**

It is concluded that high level production and intrinsic slow HBsAg VLP assembly kinetics are leading to retention and accumulation of the antigen in the endoplasmic reticulum where it assembles at least partly into defined lamellar structures. Further transport of HBsAg to the Golgi apparatus is impaired thus leading to secretory pathway disfunction and the formation of an extended endoplasmic reticulum which bulges into irregular cloud-shaped formations. As VLPs were not found within the cells it is concluded that the VLP assembly process must take place during down-stream processing after detergent-mediated disassembly of HBsAg lamellas and subsequent reassembly of HBsAg into spherical VLPs.

## Background

Unlike many other vaccines against virus-caused diseases, a single viral polypeptide is sufficient to elicit a protecting immune response against Hepatitis B infection [1].

However, the polypeptide needs to assemble into spherical particles, so-called virus-like particles (VLPs), to initiate the required protective immune response [2]. In HBV infected individuals, viral surface proteins are produced in large excess in liver cells over the amount needed for virus assembly and are secreted as a mixture of spherical particles and tubular forms. Thus, in the serum of HBV infected patients, intact viruses (Dane particles) but also "empty" spherical particles and tubular forms consisting of the surface proteins of the Hepatitis B virus are found [3,4].

The first commercial vaccine was obtained from the plasma of asymptomatic virus carriers which contained HBsAg assembled into 22-nm spheres [1]. Safety issues as well as economical motives were driving the development of a vaccine utilizing modern DNA technology and, thus HBsAg became the first recombinant protein-based vaccine, approved in 1986 by the Federal Drug Administration (USA), for human vaccination [5]. Initially, the yeast *Saccharomayces cerevisiae *was employed for commercial production of HBsAg from the viral gene encoding the 226 amino acids protein, as initial attempts to use *E. coli *based expression systems did not lead to the formation of immunoprotective material [6,7] and mammalian based expression systems turned out to be too costly for vaccine production [1]. The human plasma and recombinant mammalian cell derived vaccines are glycosylated [1,8]. In contrast, yeast derived HBsAg is not glycosylated neither from *S. cerevisiae *[9] nor from the methylotrophic yeasts *Hansenula polymorpha *[10] and *Pichia pastoris *[11].

Expression of the native Hepatitis B virus S gene in stably transfected mammalian cells leads to the secretion of HBsAg particles [12,13], however, attempts to achieve secretion of HBsAg in yeast have been unsuccessful. When the native viral gene is expressed in yeast, the protein is retained within the cells, but also efforts to enforce secretion in yeast by utilizing potent yeast or other eukaryotic secretion signals only lead to negligible amounts of secreted HBsAg with *S. cerevisiae *[8,14] or *P. pastoris *[15].

Despite wide-spread claims of intracellular VLP formation in *S. cerevisiae *[9,16,17] and the methylotrophic yeasts *H. polymorpha *[10] and *P. pastoris *[11], no clear evidence has been presented so far that particle assembly occurs within the yeast cells. Electron microscopic proofs of VLP formation were always presented for purified material which was obtained from detergent-treated cell lysates after several down-stream processing steps [9,10,18]. In contrast to those claims of intracellular VLP assembly, it has been also speculated that VLP assembly from yeast produced material might occur during down-stream processing [19,20]. Thus, surprisingly, the question, if the HBsAg protein assembles in vivo inside the yeast cell into VLPs or later in vitro during down-stream processing, has no clear answer yet.

In this work we provide evidence that in *Pichia pastoris*, expression of the native viral HBsAg gene leads to translocation of the protein into the endoplasmic reticulum (ER) where it assembles, at least partly, into defined multi-layered lamellar structures. The HBsAg is retained within the ER or perinuclear space which bulges into cloud-shaped irregular formations. Despite intensive search we could not find any evidence for the presence of VLPs within the cells and thus conclude that VLP assembly must occur after cell breakage during subsequent down-stream processing.

## Results

The production of HBsAg was carried out in high-cell density fed-batch cultures with recombinant *P. pastoris *GS115, using the methanol inducible *AOX1 *expression system [18]. Cells were first grown in a batch procedure on defined medium with glycerol as carbon substrate. After depletion of glycerol, the production of HBsAg was induced by the addition of methanol to a final concentration of 6 g L^-1^. This methanol concentration was kept constant for the rest of the cultivation by continuous methanol feeding. During growth on methanol, intracellular accumulation of HBsAg was observed reaching a maximum concentration of 7 g L^-1 ^which corresponds to approximately 70 mg HBsAg per g cell dry mass, with 30 to 40% of it being "soluble" and competent for assembly into VLPs [18].

**Figure 1:**
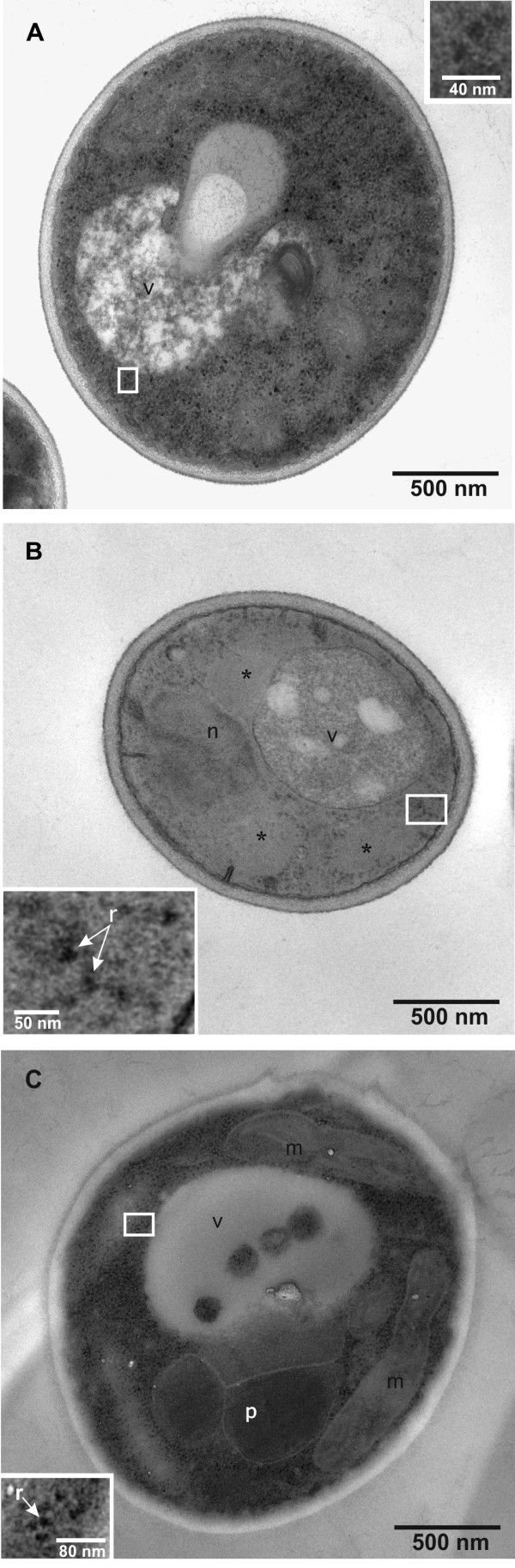
**Images of recombinant *P. pastoris *growing on glycerol and methanol**. Transmission electron microscopy of ultrathin sectioned cells of *P. pastoris *GS115 growing on (A) glycerol and (B) after 151 h growth on methanol for induction of HBsAg production. (C) Image of *P. pastoris *X-33 after 96 h growth on methanol for induction of secretory insulin precursor production [38]. Abbreviations: n, nucleus; v, vacuole; m, mitochondrion; r, ribosome; p, peroxisome; *, 'irregular cloud-shaped area'. The insets show close-ups of ribosomes from marked areas of respective images.

**Figure 2:**
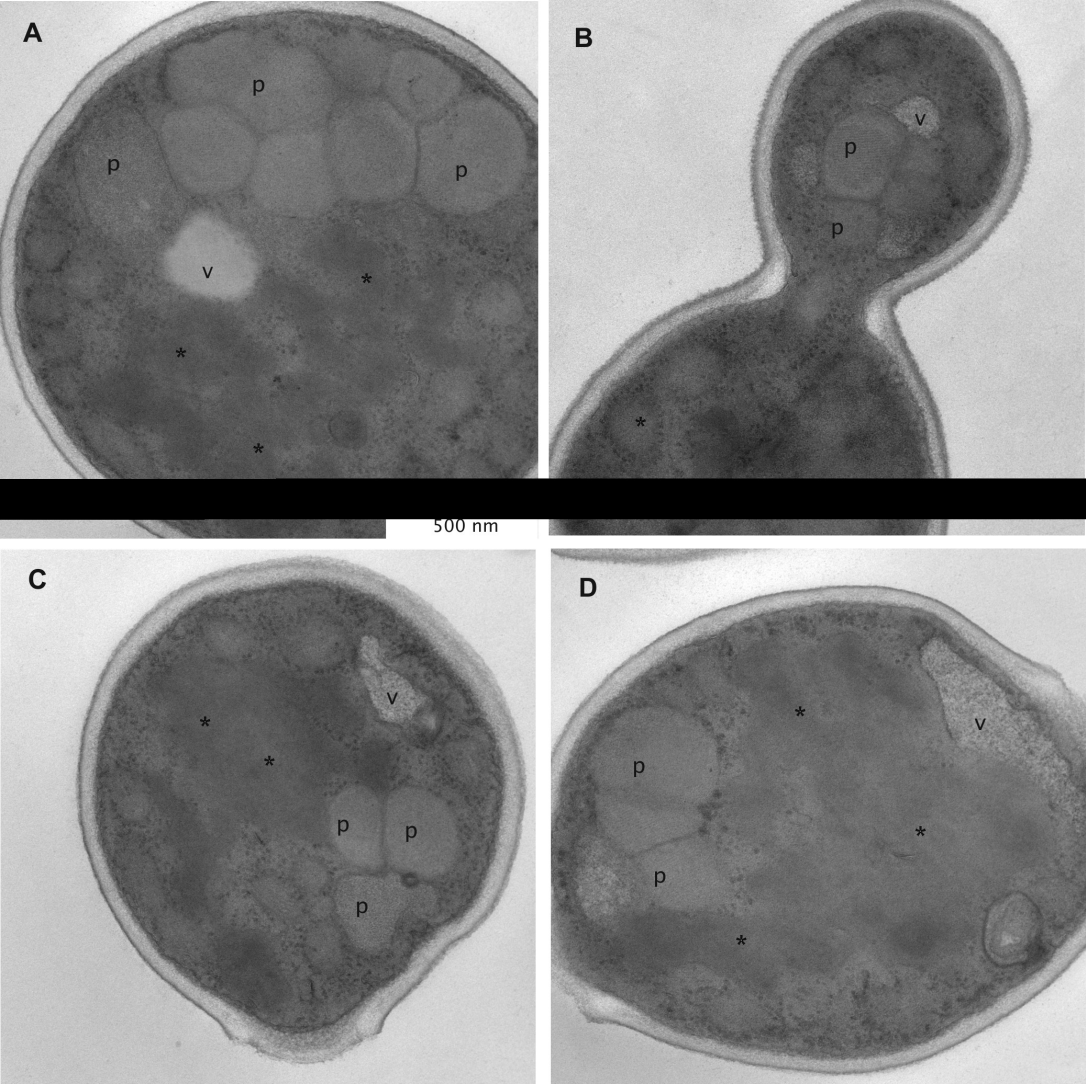
**Images of recombinant *P. pastoris *producing HBsAg during growth on methanol**. (A-D) Representative transmission electron micrographs of ultrathin sectioned cells of *P. pastoris *GS115 grown for 151 h on methanol as described in the Materials and Methods section. Abbreviations as specified in Figure 1.

**Figure 3:**
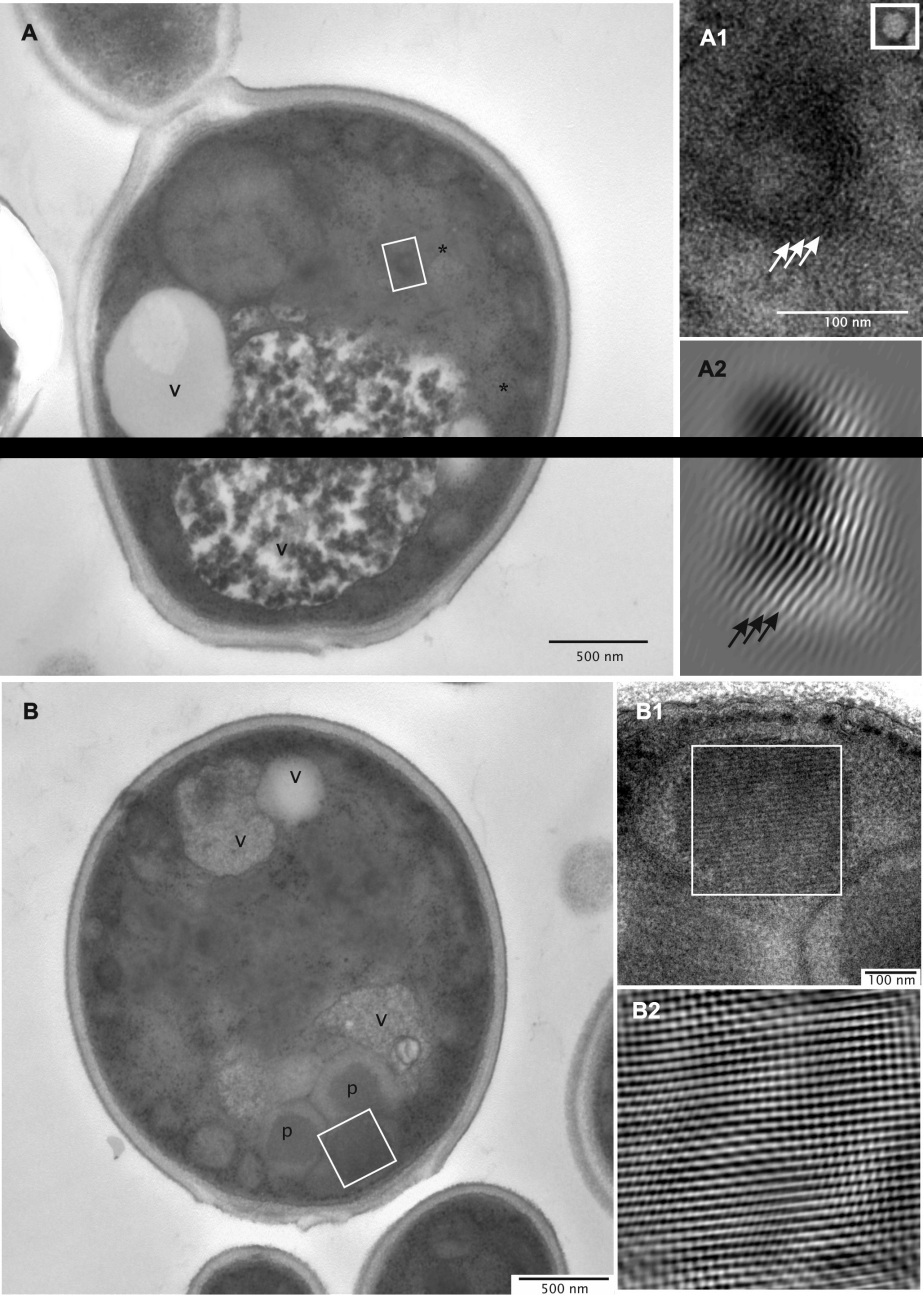
**Close-up views of *P. pastoris *GS115 producing HBsAg**. Transmission electron micrographs of single cells are shown in A and B. A1 is enlarged from the boxed area of A localized in the 'irregular cloud-shaped area' (*), and the square-boxed insert of A1 shows a negatively stained reconstructed VLP with the same scale as in A1. A2 represents the corresponding inverse fast Fourier transformation (iFFT) of area A1. The 6 nm layer-spacing is indicated by arrows in A1 and A2. B1 is enlarged from boxed area in B; B2 represents the corresponding inverse fast Fourier transformation (iFFT) of area B1. Abbreviations as specified in Figure 1.

To determine the location and appearance of the Hepatitis B surface antigen in overproducing cells, electron microscopic studies in combination with immunogold labeling were carried out. Cells growing on glycerol and cells after induction of HBsAg synthesis through methanol feeding were first subjected to transmission electron microscopy for ultrastructural analysis (Figure 1A and 1B, respectively). The ultrastructure of the cells changed substantially after exposure to methanol. In the cytosol of HBsAg-producing cells large irregular cloud-shaped areas of medium electron density became apparent (Figure 1B). These morphological features were absent in cells growing on glycerol (Figure 1A) and also absent in cells producing insulin precursor as secreted protein after exposure to methanol (Figure 1C). Both cells producing either HBsAg or insulin precursor on methanol contained microbodies (peroxisomes) with internal crystal-like structures (Figures 1C, Figure 2, Figure 3B). These peroxisomes are typical for methylotrophic yeast cells when growing on methanol [21,22] and mainly contain enzymes necessary for the breakdown of carbon sources such as alcohols or fatty acids (e.g. alcohol oxidases, catalases, acetyl-CoA oxidases) [23-25].

**Figure 4:**
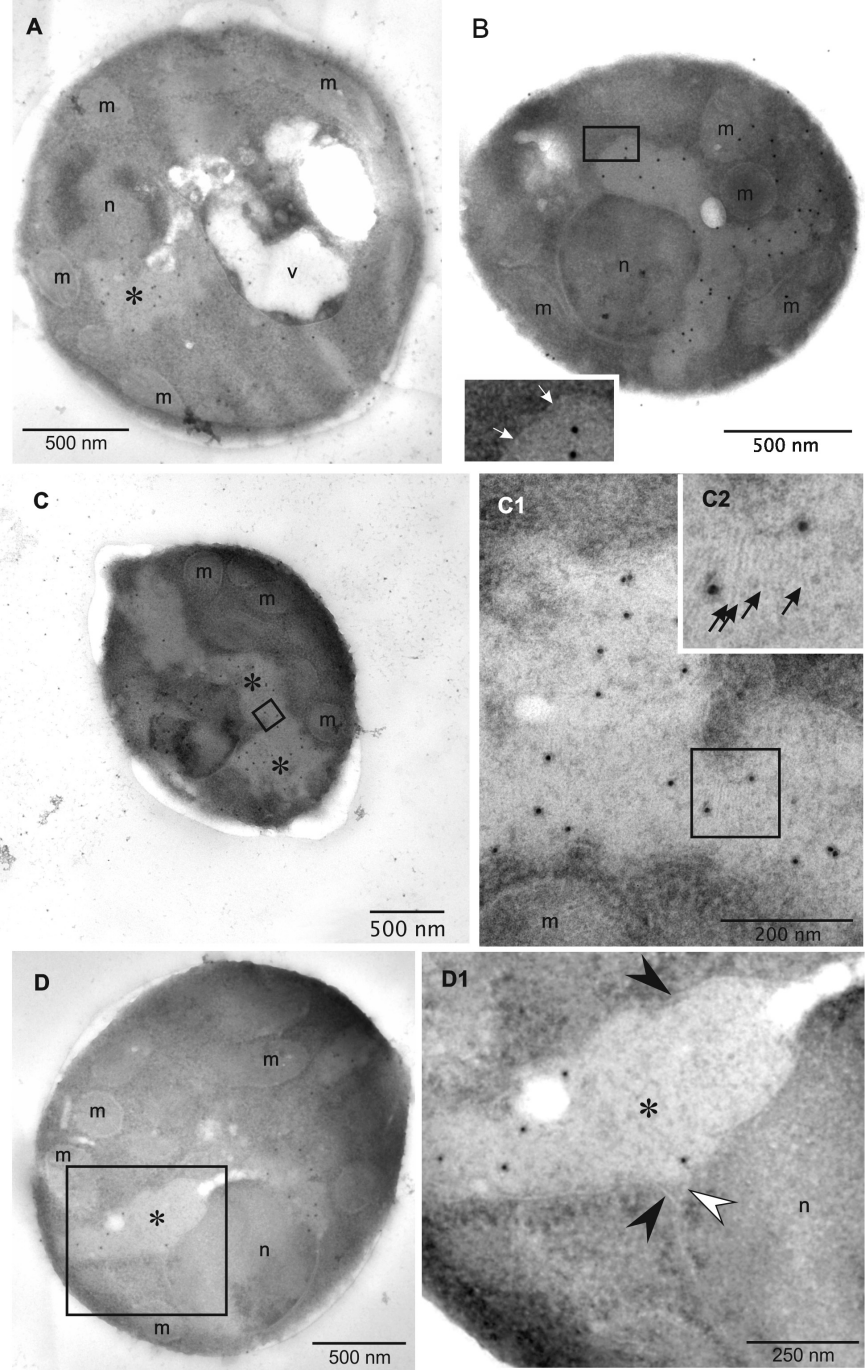
**Immunogold labeling and close-up views of *P. pastoris *GS115 producing HBsAg**. The antigen was labeled with rabbit antibodies specific for HBsAg and is shown by dark Protein A-gold conjugates, localized in the brighter 'irregular cloud-shaped area' (*). (A-D) Representative images of cells grown on methanol as described in the Materials and Methods section. The insert in B represents a close-up view of the boxed area in B with the white arrows pointing to the cytoplasmic side of the membrane surrounding the 'irregular cloud-shaped area' (*). C1 and C2 represent sequential enlargements of the corresponding boxed area in C. Black arrows (C2) point to the lamellar striation within the labeled 'irregular cloud-shaped area' (*). Black and white arrowheads in D1 point to the cytosolic and nucleic side of the nuclear double membrane, respectively. Abbreviations as specified in Figure 1

However, a comprehensive search of electron micrographs of *P. pastoris *cells containing high levels of HBsAg for the presence of intracellular VLPs remained negative (Figures 1B, 2, 3, and 4). Circular, electron dense structures which might be misjudged as VLPs, found evenly distributed in the cytosol and also clustered on membranes, were identified as ribosomes (see also inserts in Figures 1A-C). These "particles" were also visible in cells not exposed to methanol and thus in the absence of HBsAg production and in cells secreting insulin precursor during growth on methanol.

The ultrastructural appearance of HBsAg-producing cells appeared quite diverse, but all cells contained these large irregular cloud-shaped areas of medium electron density in addition to the other typical yeast organelles such as nuclei, mitochondria and vacuoles (Figures 1B, 2, 3). These structures were consistently observed in cells which produced high levels of intracellular HBsAg but not in non-producing cells. Upon closer inspection a multi-layered, lamellar organization within these areas became apparent (Figure 3A1). For more clarity, an inverse fast Fourier transformation (iFFT) was carried out which revealed a defined spacing of app. 6 nm between the layers (Figure 3A2). These multi-layered lamellas were consistently observed in the HBsAg producing cells, but we could not find any indication for the presence of HBsAg VLPs (22 nm diameter) which should be clearly visible at this magnification (Figure 3A1).

For comparison, close-ups and iFFTs of the typical internal crystal-like structures of peroxisomes reveal a line-spacing and thus a layer thickness of 10-11 nm (Figure 3B) characteristic of well-ordered protein layers composed of highly symmetrical building blocks [26,27]. In contrast to the crystal-like peroxisomal core regions with its straight layer lines (Figure 3B1 and 3B2), layers in the electron-dense cloud-shaped areas of the HBsAg producing cells appear bent and show distinct curvature of layer stacks (Figure 3A1 and 3A2) reflecting a different arrangement of the building block within the layer planes indicative of its potential to form circular vesicle structures.

**Figure 5:**
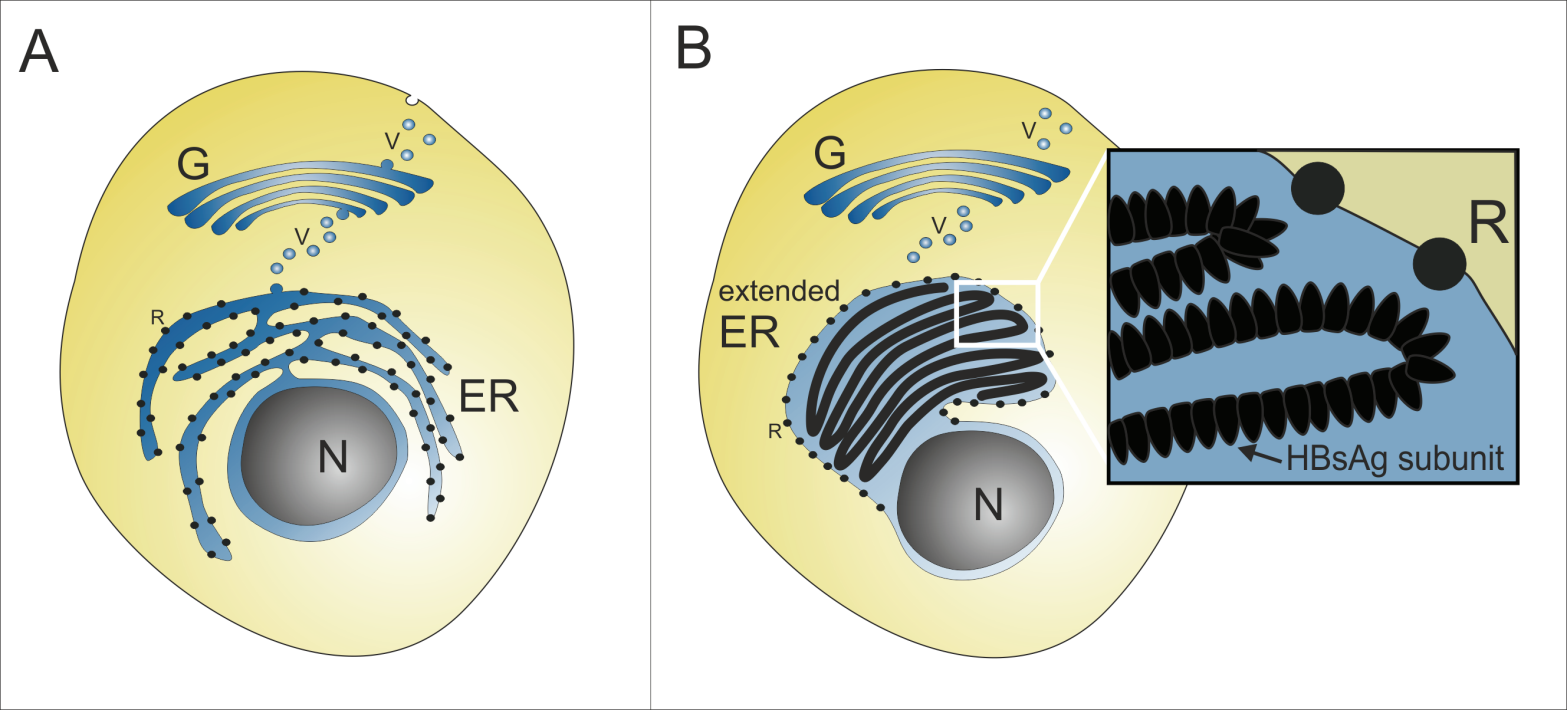
**Conventional protein secretion and HBsAg production in *P. pastoris***. Cartoon displaying (A) the conventional secretory protein production pathway and (B) the HBsAg production and translocation pathway. N, nucleus; ER, endoplasmic reticulum; G, Golgi apparatus; V, secretory vesicle; R, ribosome; HBsAg, Hepatitis B surface antigen.

**Figure 6:**
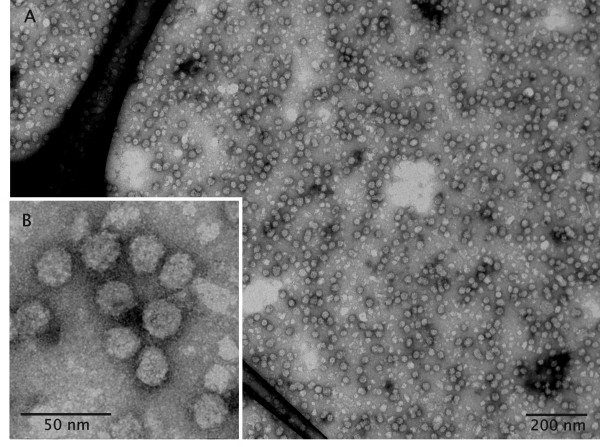
**Purified VLPs composed of HBsAg**. Transmission electron microscopy of negatively stained HBsAg VLPs after purification from detergent-treated cell lysate. Cells were collected after 170 h of growth on methanol and the HBsAg purified from the cell lysate as described in the Materials and Methods section. (A) Survey of negatively stained HBsAg VLPs and (B) close-up view revealing particles of uniform diameter (~22 nm) and approximate icosahedral contour.

Immunogold labeling of HBsAg producing cells with diluted polyclonal antibodies specific for HBsAg revealed selective labeling of the irregular shaped areas containing the multi-layered, lamellar structures strongly suggesting that the HBsAg protein assembles into these lamellas (Figure 4). Theoretical calculations of the size of a single HBsAg molecule (26 kDa) indicate a diameter of 3 nm in case the molecule would adopt a spherical shape [28,29]. However, structural studies on purified HBsAg particles revealed that the smallest building blocks are HBsAg dimers in which the monomers are present in an extended conformation [4,30]. Thus, the interlayer spacing of 6 nm suggests that these lamellas represent HBsAg monolayers (Figures 3A and 4C). Further close-up views also show that these structures are not dispersed in the cytosol but arise in the perinuclear space between the cytosolic and nucleic part of the nuclear double membrane leading to the formation of an extended ER (Figure 4D). These findings clearly show that the protein is translocated into the endoplasmic reticulum but is not further processed in the secretory pathway. High level production concomitant to ER retention leads to the formation of an extended ER which bulges into cloud-shaped formations (see cartoon Figure 5). However, electron microscopic investigation of HBsAg purified from detergent treated cell lysates shows that the protein produced by *P. pastoris *can form VLPs (Figure 6). The absence of VLPs in the intact cells and the presence of VLPs in the final purified protein clearly show that the VLP assembly process does not occur in vivo but in vitro during down-stream processing and purification.

## Discussion

We could not find any indication that the assembly of the HBsAg polypeptide into VLPs occurs inside of *P. pastoris*. Instead, we provide evidence that the protein accumulates in an extended ER where it is found to assemble at least partly into multi-layered, lamellar structures. The layer spacing suggests the presence of HBsAg monolayers and clearly shows that HBsAg assembles into defined multimeric structures indicative of a well-ordered monomer fold. The extended ER may also contain aggregated HBsAg as we did not see a comprehensive filling of the extended ER with these lamellar structures. However, it should also be noted that the visibility of these lamellar structures strongly depends on their parallel orientation relative to the electron beam. Translocation and retention of HBsAg in the ER of the yeast cell appears to be an intrinsic property of this very hydrophobic protein which has long stretches of connected hydrophobic amino acids.

The native viral HBsAg protein contains an N-terminal secretion signal which directs this protein in mammalian cells to the ER without concomitant cleavage of the N-terminal sequence [31]. This secretion signal is obviously also recognized by *P. pastoris*; the HBsAg is translocated into the lumen of the ER. In *P. pastoris*, however, the protein is retained in the ER and not further processed in the secretory pathway (Figure 5). In contrast, in mammalian cells which are stably transfected with the *HBsAg *gene, HBsAg particles are processed through the secretory pathway and secreted into the extracellular environment [12,13]. For mammalian cells, convincing evidence based on ultrastructural analysis has been presented which clearly shows that HBsAg assembles into VLPs in the ER [12]. However, it has also been shown that secretion of HBsAg from recombinant mammalian cells is a very slow process with a long half life (~5 h) which is characteristic for HBsAg, as other viral envelope proteins are secreted with normal kinetics (~30 min) [13]. These findings indicate an intrinsic slow assembly process of HBsAg into VLPs.

Compared to mammalian cells, protein synthesis rates are clearly faster in yeast cells in particular when the respective gene is under control of a strong promoter. When VLP assembly kinetics are slow compared to rates of protein synthesis and translocation into the ER, molecular crowding in the ER will interfere with the assembly of the protein into VLPs. This impairs further transport to the Golgi apparatus thus leading to secretory pathway dysfunction. Apparently, molecular crowding results in "mis-assembly" of HBsAg into the observed multi-lamellar structures in an extended ER which bulges into cloud-shaped irregular formations. Similarly, intracellular retention of HBsAg and failure of the protein to assemble into VLPs has also been reported from mammalian cells under conditions of elevated production [32]. Moreover, plant-cell derived HBsAg is not secreted but accumulates in the ER in tubular structures, differing substantially in appearance from the uniform size distribution of VLPs [33,34]. Tubular forms are not only formed in the ER of recombinant expression systems but are even found in the sera of chronic HBV-infected humans [3,4]. Thus, productive assembly pathways of folded HBsAg monomers seem to include the formation of VLPs but also the formation of tubular or even lamellar structures. Under conditions of intense production multi-lamellar structures will be favored as the HBsAg VLP assembly process is slow. However, our results indicate that these structures can be transformed into energetically more favorable VLPs under appropriate conditions. As we could not detect any VLPs within *P. pastoris*, we conclude that the VLP assembly process from yeast-derived HBsAg must take place during down-stream processing and purification.

## Methods

### Strains and growth conditions

The *P. pastoris *strain GS115 carrying 8-copies of the *HBsAg *structural gene under the control of the *AOX1 *promoter has been described before [35]. The *HBsAg *gene was inserted in between the promoter and transcription termination regulatory elements without using yeast-derived secretory signals. Cells were grown on defined medium in a fed-batch procedure as described [18]. High-level production of HBsAg was initiated after batch growth on glycerol through the addition of methanol to a final concentration of 6 g L^-1^. This methanol concentration was kept constant by continuous methanol feeding throughout the entire production phase. Process analytics and the determination of soluble and insoluble HBsAg produced by *P. pastoris *have been described previously [18].

### Purification of HBsAg

Cells from one liter culture broth were collected by centrifugation (Sorvall RC5BPlus, SLA 3000 rotor) at 4°C and 6,000 rpm for 15 min, resuspended in 25 mmol L^-1 ^phosphate buffer (pH 8.0) and re-centrifuged. The cell pellet was resuspended in ice-cold lysis buffer [25 mmol L^-1 ^phosphate buffer (pH 8), 5 mmol L^-1 ^EDTA, 0.6% (v/v) Tween-20] and the pre-cooled cell suspension partly disrupted by high pressure homogenization (Gaulin Lab 60, APV Gaulin, Germany) with three cycles at 600 bar and ~4°C. NaCl (5 mol L^-1^) was slowly added within 30 min to the cell lysate to a final concentration of 0.5 mol L^-1 ^followed by the addition of polyethylene glycol 6000 (50% w/v) to a final concentration of 5% (w/v). Precipitation was allowed to occur for 12-16 h at 4°C and the suspension was then clarified by centrifugation at 4°C and 6000 rpm for 15 min. The resultant supernatant was mixed with Aerosil 380 (Evonik, Hanau, Germany) which was pre-equilibrated in 25 mmol L^-1 ^sodium phosphate buffer (pH 7.2), 0.5 mol L^-1 ^NaCl (0.13 g of dry Aerosil 380 per gram of initial wet biomass). The suspension was stirred for 4 h at 4°C and centrifuged at 4°C and 6000 rpm for 15 minutes. The pellet was washed twice with 25 mmol L^-1 ^phosphate buffer (pH 7.2), and centrifuged as above. The resultant pellet was resuspended in 100 mmol L^-1 ^sodium carbonate-bi-carbonate buffer (pH 10.8), 1.2 mol L^-1 ^urea and kept at 37°C for 12 h with stirring. This suspension was then centrifuged at 25°C and 10,000 rpm for 60 minutes and the supernatant clarified by vacuum-filtration (0.45 μm). The supernatant pH was adjusted to pH 8.0 and proteins allowed to bind to the DEAE sepharose FF (Amersham Pharmacia Biotech, Sweden) pre-equilibrated with 100 mmol L^-1 ^sodium carbonate-bi-carbonate buffer (pH 8.0) using an XK column (Amersham Pharmacia Biotech, Sweden). After sample loading, the column was washed with 50 mmol L^-1 ^Tris-HCl (pH 8.0) until the absorbance at 280 nm returned to baseline. Elution of bound HBsAg was carried out with 50 mmol L^-1 ^Tris-HCl buffer (pH 8), 0.5 mol L^-1 ^NaCl. Protein containing fractions (absorbance at 280 nm) were pooled and mixed with caesium chloride (final concentration: 1.2 g mL^-1 ^CsCl). This mixture was layered on top of an ultracentrifuge tube (T-865B tube, SORVALL) containing a CsCl solution with a density of 1.2 g mL^-1^(density of HBsAg VLPs ~1.18 g mL^-1^) and centrifuged at 22°C and 50,000 rpm for 12 h (TV-865B rotor, SORVALL). The protein containing fractions of the ultraeluate were treated with KSCN at a final concentration of 1.2 mol L^-1 ^and this mixture incubated at 37°C for 5 h in an orbital shaker (Multitron II, Infors AG, Germany), then dialyzed against phosphate buffered saline (PBS), sterile filtered, and stored as a concentrated solution of 500 μg mL^-1 ^HBsAg VLPs.

### Electron microscopy

#### Fixation and embedding

Samples from bioreactor cultivations were immediately fixed at ambient temperature in 2.5% (v/v) glutardialdehyde in 20 mmol L^-1 ^HEPES buffer (pH 7.1) for 30 min. Cells were stored until embedment for several days at 4°C. For ultrastructural analysis cells were immobilized in 1% (w/v) agar and further fixed in 1% (w/v) osmiumtetroxide in 75 mmol L^-1 ^cacodylate buffer (pH 7.2). Cells were dehydrated on ice in an ethanol series, stained with 1% (w/v) uranylacetate in 70% (v/v) ethanol (for methacrylic LR-Gold embedding this staining step was omitted) and finally infiltrated with epoxy resin [36]. Cells were polymerized at 70°C for 8 hours. Ultrathin sections (90 nm) were cut with a diamond knife using an ultramicrotome (Leica, Wien, Austria), picked with Formvar-coated 300 mesh copper grids and poststained with uranyl acetate and lead citrate as described previously [37].

Cells used for immunocytochemical analysis were fixed in 0.5% (v/v) glutardialdehyde, 3.5% (w/v) paraformaldehyde in 20 mmol L^-1 ^HEPES buffer (pH 7) for 30 min at 20°C and kept at 4°C until further treatment for several days in a refrigerator. For embedding, cells were immobilized in 1% (w/v) agar, dehydrated in an ethanol series and infiltrated in metacrylic LR-Gold resin. Polymerization was carried out with 0.1% (w/v) benzil added at ambient temperature for 60 hours.

#### Immunocytochemical treatment for antigen detection (Immunogold labeling)

The ultrathin sections (90 nm) were picked with 300 mesh Ni-Butvar grids and layered on top of 30 μl commercially available rabbit polyclonal anti-HBsAg antibodies (Cat. No, BP 2029P, Acris Antibodies GmbH, Herford, Germany) in PBS (pH 7, 1:200 dilution) and incubated at 4°C overnight (sections on pure PBS buffer were used as blank control). The HBsAg antibody was used directly without immuno-affinity purification on HBsAg columns, however specificity against HBsAg was confirmed by Western blot using cell samples with and without HBsAg and by immunogold labeling of cells producing HBsAg and control cells not producing HBsAg (data not shown). After incubation with HBsAg antibodies, grids were washed 3 times in PBS for 10 min at ambient temperature. Labeling was done with protein G-gold 10 nm (Plano, Wetzlar, Germany), which binds specifically to the Fc part of IgG antibodies, diluted 1:200 with PEG-PBS (0.5 mg mL^-1 ^polyethylene glycol 20,000 in PBS, pH 7) for 30 min at ambient temperature. Grids were washed twice for 5 min with 10 mmol L^-1 ^HEPES (pH 7), 0.03% (v/v) Tween 20, followed by two additional washing steps for 5 min with 10 mmol L^-1 ^HEPES (pH 7) and one step with 10 mmol L^-1 ^HEPES (pH 7), 1 mmol L^-1 ^EDTA for 5 min. The grids were then washed with 20 mmol L^-1 ^Tris-HCl (pH 7), 5 mmol L^-1 ^EDTA-Na_4 _for two minutes and stained with 2% (w/v) uranyl acetate for 5 min. Finally, the grids were jet-washed with water and the residual water was removed by filter paper and air-drying.

Ultrathin sections were analyzed with an energy-filtered transmission electron microscope (CEM 902, Zeiss, Oberkochen, Germany) in the elastic bright-field mode and images were captured with a 1 × 1 k CCD camera (Proscan, Scheuring, Germany). Images of cells prepared for ultrastructural analysis or immunolabeling appear different as sample preparations followed different protocols.

#### Image processing

Ultrastructural analysis for noise reduction was carried out using fast Fourier transformation (FFT) and inverse fast Fourier transformation (iFFT) by CRISP software application (CRISP ver. 2.1; Calidris, Sollentuna, Sweden).

## Competing interests

The authors declare that they have no competing interests.

## Authors' contributions

HL carried out the electron microscopic studies and drafted the results section. CG purified the HBsAg. Both, CG and AA were involved in EM sample generation and NK in the initial outline of the project. UR conceived and directed the study and prepared the final manuscript. All authors read and approved the final manuscript.
